# Phosphate Dosing in Drinking Water Distribution Systems Promotes Changes in Biofilm Structure and Functional Genetic Diversity

**DOI:** 10.3389/fmicb.2020.599091

**Published:** 2020-12-17

**Authors:** Esther Rosales, Gonzalo Del Olmo, Carolina Calero Preciado, Isabel Douterelo

**Affiliations:** Department of Civil and Structural Engineering, The University of Sheffield, Sheffield, United Kingdom

**Keywords:** biofilms, phosphate, drinking water, distribution networks, whole metagenomics

## Abstract

Water utilities treat drinking water by adding phosphate to prevent metal dissolution from water pipe work systems and particularly lead poisoning. Phosphate can be a limiting nutrient for microbial biofilms in DWDS, yet its effects on these microbial consortia are not well understood. This research presents results from phosphate dosing experiments using a real scale chlorinated DWDS, comparing standard phosphate concentrations of United Kingdom drinking water (1 mgP/L) with a double dose (2 mgP/L) commonly used in plumbosolvency treatment. Biofilm development during phosphate treatment experiments was monitored using a holistic approach by combining metagenomics analysis, flow cytometry and SEM characterisation. The increase of phosphate levels in drinking water, reduced biofilm cell numbers and promoted the presence of poorly distributed biofilms on inner pipe surfaces. Metagenomics analysis using genetic markers (16S rRNA and ITS2) showed that phosphate influenced biofilm community structure, particularly fungal composition. Whole metagenome sequencing showed that phosphate enrichment favoured the presence of sequencing reads associated to ATPases, ion transporters and DNA-interacting proteins, whilst reads associated to nitrogen metabolism were predominant in control samples. This research brings new knowledge regarding the influence of phosphate treatment on the composition and structure of biofilms within DWDS, and the implications that this might have for the management of these systems.

## Introduction

Metal leaching from water pipes, mostly lead dissolution, is the main cause of drinking water contamination in many countries, thus endangering public health (Gordon and Hutchinson, 1994; Edwards, 2014). Water companies treat water by adding phosphate to prevent the corrosion and metal leaching occurring in Drinking Water Distribution Systems (DWDS) (Mavropoulos et al., 2004; Gooddy et al., 2015). Different inorganic phosphate forms can be supplied into the water but, usually, water companies add orthophosphate (Berger et al., 1992) to reduce the solubility of metals used in pipe work systems such as lead, copper and iron (Schock, 1989; Schock and Clement, 1996; Lytle et al., 2003; Mavropoulos et al., 2004). The affinity of orthophosphate ions for hydrated oxide particles leads to interactions between pipe surface metals and phosphate anions. These interactions prevent corroded particles such as iron or lead from dissolving in the drinking water and limit the corrosion process in pipes (Persson et al., 1996; Gooddy et al., 2015).

Despite phosphate has proven to be effective against metal piping dissolution (Mcneill and Edwards, 2001; McNeill and Edwards, 2002; Hayes et al., 2008), limited knowledge exist on its effect on the microbial ecology of DWDS, and in particular on biofilms. In DWDS, microorganism not only inhabit bulk water, they can be found forming biofilms on the inner pipe surfaces (Douterelo et al., 2013), representing more than 98% of biomass within DWDS (Liu et al., 2014). Biofilms are diverse microbial consortia composed of bacteria, fungi, archaea, viruses, and protists (Hull et al., 2019; Mathieu et al., 2019), which are embedded in a matrix of extracellular polymeric substances (EPS) (Donlan and Costerton, 2002). These organisms had complex interactions with the pipe environment and between them and are responsible for causing several problems in DWDS. Biofilms in DWDS have been associated with metal bio-corrosion (Szewzyk et al., 2000; Bremer et al., 2001; Beech and Sunner, 2004; Douterelo et al., 2013), water discolouration events (Lau and Ashbolt, 2009; Douterelo et al., 2013) and pathogens appearance (Lau and Ashbolt, 2009).

Even though it is considered that the principal limiting nutrient in DWDS is carbon, phosphorus can also limit microbial growth in aquatic ecosystems (Miettinen et al., 1997; Sathasivan et al., 1997). Previous studies on the effect of phosphate in DWDS present several limitations, since most of them were performed under *in vitro* conditions and were directed to evaluate planktonic microorganisms and/or biofilms developed in bioreactors using materials such as polycarbonate and cast iron (Miettinen et al., 1997; Sathasivan et al., 1997; Keinänen et al., 2002; Kasahara et al., 2004). In addition, those studies focused on investigating bacterial communities, yielded contradictory results about the effect of phosphate on these microorganisms. On the one hand, several researchers found that phosphate favoured bacterial growth (Miettinen et al., 1997; Sathasivan et al., 1997), increased bacterial cell numbers (Kasahara et al., 2004; Fang et al., 2010), promoted biofilm viability (Rubulis and Juhna, 2007) and increased Gram-negative bacterial content (Kasahara et al., 2004) and diversity (Jang et al., 2012; Payne et al., 2016). On the other hand, other researchers indicated that phosphate did not affect bacterial density (Berger et al., 1992; Gouider et al., 2009) and a decrease of bacteria was observed when this chemical was added (Appenzeller et al., 2001). It can be suggested that these contradictory results might be due to the different environmental/laboratory conditions where each experiment was carried out (Appenzeller et al., 2001), making the comparison of results and drawing conclusions difficult.

This study used real scale and representative hydraulic conditions of DWDS to provide new insights on the process of biofilm formation in networks treated with phosphate. This new knowledge can inform the optimisation of phosphate dosing strategies used by water utilities to prevent plumbosolvency.

## Materials and Methods

### Experimental Facility and Operating Conditions

**FIGURE 1:**
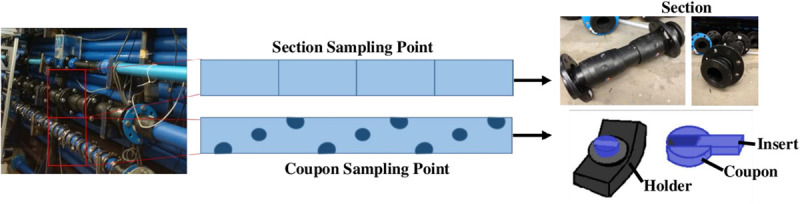
Section and Coupon sampling point in the DWDS facility and details of both biofilm support kind.

A chlorinated real scale DWDS (Figure 1) test facility was used to test the effect of phosphate dosing on biofilm development. The facility consists of 3 independent loops made of HDPE as described in Douterelo et al. (2018b). In this experiment, only two loops of the test facility were used: one loop was maintained as Control, with no phosphate addition, and the other loop was set to double the phosphate concentration (2 mg/L) (Phosphate Treatment). The water running through the system came from the local drinking water supply, this water is treated with chlorine and has an average phosphate concentration of 1 mg/L. The phosphate commercial compound used to treat the water was similar to that dosed by the local water utility and consisted of sodium dihydrogenorthophosphate, Monosodium Phosphate 32%, (Airedale Chemicals, Keighley, United Kingdom). Phosphate was constantly dosed in the tank of one of the loops by means of a peristaltic pump (Peristaltic Watson and Marlow 505 pump, Fluid Technical Group, Cornwall, United Kingdom). The hydraulic regime used during the duration of the experiment was a low varied flow regime, ranging from 0.2 to 0.5 L/s, based on average daily patterns observed in real DWDS in the United Kingdom (Husband et al., 2008). This regime has a 24-h cycle, which was repeated for a growth phase of 28 days. The temperature of the facility was set to 16°C; this is representative of average spring and summer temperatures in United Kingdom DWDS, thus accurate for real systems but providing maximum representative levels of microbial activity.

Before the start of the experiment, the facility was disinfected with 20 mg/L of RODOLITE H (RODOL Ltd., Liverpool, United Kingdom) which is a solution of sodium hypochlorite with less than 16% free available chlorine. Then the system was run at maximum flow rate (4.2 L/s) for 24 h and flushed afterwards at the maximum flow rate with fresh drinking water until the levels of chlorine were similar to those of the local tap water.

Two different supports for biofilm development were used (Figure 1): sterile Pennine Water Group (PWG) coupons (Deines et al., 2010) which were arbitrarily fitted along and around the sample length of each pipe loop and tubular sections made of HDPE, with a length of 0.5 m and an internal diameter of 79.3 mm, which were installed in another length of pipe in each loop (Figure 1). The PWG coupons and sections were installed before RODOLITE H administration, so they were disinfected within the system. The use of PWG coupons allowed for the flow cytometry and SEM analysis and provided DNA for the analysis of marker genes (bacteria and fungi), whilst the sections were used for flow cytometry analysis and to obtain the necessary concentration of DNA from biofilms for whole metagenomics analysis.

### Sampling of Biofilms

To study biofilm communities within the system, PWG coupons were collected weekly (days 0, 7, 14, 21, and 28) and sections were extracted on day 28 (final day of the experiment). Six PWG coupons were removed from each loop per day of sampling and six removable sections were taken per loop at day 28. In total 30 biofilm samples were acquired from the outer part of PWG coupons as described in Deines et al. (2010) and the inner part was used for flow cytometry and SEM analysis. In addition, another 12 biofilms samples for DNA extraction and flow cytometry were removed from sections, following the same methodology as Deines et al. (2010). The removed biofilms were suspended in a solution of Phosphate-buffered saline (PBS) (pH 7), solutions were filtered through a sterile filter (0.22 μm MCE Membrane MF-Millipore, United Kingdom), one per sample. Filters were then kept in the dark and at −20°C for subsequent DNA extraction.

### Water Physico-Chemical Analysis

Several physico-chemical parameters, were analysed by collecting discrete water samples on each sampling day. Triplicate measurements were obtained for each parameter and the average of the three replicates was calculated as showed in results (Table 1). Free chlorine was measured using a DR/2010 spectrophotometer (Hach, United Kingdom). Measurements of temperature and pH were made using a Portable pH and ORP Meter H1991003 (Hanna Instruments, United Kingdom). Water samples for total iron, manganese, total organic carbon (TOC), orthophosphate and phosphorus were sent to an accredited drinking water laboratory; ALS Environmental Ltd. (Coventry, United Kingdom) for analysis.

**TABLE 1:** Physico-chemical analyses in the different sampling days of every experimental case.

	**Day 0**		**Day 7**		**Day 14**		**Day 21**		**Day 28**	
	**Control**	**Phosphate treatment**	**Control**	**Phosphate treatment**	**Control**	**Phosphate treatment**	**Control**	**Phosphate treatment**	**Control**	**Phosphate treatment**
Room temperature (°C)	12.80 ± 1.06	12.80 ± 1.06	14.17 ± 0.21	14.17 ± 0.21	13.63 ± 0.35	13.63 ± 0.35	14.20 ± 0.10	14.20 ± 0.10	13.27 ± 0.25	13.27 ± 0.25
Water pH	5.30 ± 0.11	4.60 ± 0.07	6.74 ± 0.05	6.65 ± 0.11	6.36 ± 0.04	6.44 ± 0.03	6.27 ± 0.09	7.00 ± 0.20	6.82 ± 0.09	6.99 ± 0.15
Water temperature (°C)	14.20 ± 0.10	13.63 ± 0.15	14.47 ± 0.06	14.30 ± 0.10	14.23 ± 0.15	13.67 ± 0.12	14.13 ± 0.06	14.17 ± 0.06	14.13 ± 0.15	14.07 ± 0.06
Total Cl_2_ (mg/L)	0.47 ± 0.05	0.64 ± 0.02	0.35 ± 0.01	0.46 ± 0.00	0.36 ± 0.00	0.38 ± 0.07	0.35 ± 0.03	0.43 ± 0.01	0.35 ± 0.01	0.39 ± 0.01
Free Cl_2_ (mg/L)	0.41 ± 0.03	0.45 ± 0.06	0.24 ± 0.01	0.33 ± 0.01	0.24 ± 0.01	0.29 ± 0.01	0.27 ± 0.01	0.29 ± 0.01	0.25 ± 0.01	0.28 ± 0.02
Turbidity (NTU)	0.13 ± 0.05	0.17 ± 0.09	0.20 ± 0.06	0.25 ± 0.06	0.26 ± 0.01	0.22 ± 0.01	0.17 ± 0.05	0.20 ± 0.09	0.22 ± 0.05	0.19 ± 0.04
Orthophosphate as P (mg/L)*	1.10 ± 0.01	2.47 ± 0.06	1.13 ± 0.06	2.90 ± 0.35	1.07 ± 0.06	2.70 ± 0.10	1.05 ± 0.21	2.60 ± 0.00	1.17 ± 0.06	2.40 ± 0.00
Phosphorus, Total as P (mg/L)*	1.21 ± 0.17	2.60 ± 0.02	1.27 ± 0.02	2.91 ± 0.04	1.16 ± 0.03	2.68 ± 0.03	1.34 ± 0.00	2.91 ± 0.03	1.23 ± 0.02	2.50 ± 0.03
TOC as C (mg/L)*	1.47 ± 0.12	1.37 ± 0.06	1.67 ± 0.12	1.60 ± 0.10	1.23 ± 0.15	1.37 ± 0.21	1.15 ± 0.07	1.10 ± 0.10	1.10 ± 0.00	1.20 ± 0.10
Iron, Total as Fe (mg/L)*	<0.23	<0.23	<0.23	<0.23	<0.23	<0.23	<0.23	<0.23	<0.23	<0.23
Manganese, Total as Mn (mg/L)*	<0.007	<0.007	<0.007	<0.007	<0.007	<0.007	<0.007	<0.007	<0.007	<0.007

*Data provided by ALS.

### Flow Cytometry Analysis of Water and Biofilm Samples

Flow cytometry was used to study changes in biofilm and bulk water cell numbers over time. Based on previous research by Douterelo et al. (2016b), water was collected in sterile 50-ml tubes and transported in the dark and at 4 °C until analysed within 24 h of collection. SYBR^®^ Green I (Molecular Probes, Invitrogen, United Kingdom), used for staining nuclear double-stranded DNA, was diluted 1:100 with filtered dimethyl sulfoxide. 10 μl of SYBR^®^ Green I 100 × was added to 990 μl of the sample; the mix was vortexed and incubated for 15 min in the dark until measurements were carried out. The analysis was performed using a BD^TM^ LSR II Flow Cytometer System (BD Biosciences, United Kingdom). The samples were excited by a blue 488-nm laser and SYBR^®^ Green I was detected by a 505-nm long-pass and a 530/30 nm band-pass filter set. Data were processed and analysed using the BD FACSDiva software (BD Biosciences, United Kingdom).

### Scanning Electron Microscope (SEM)

Scanning electron microscopy (SEM) was used to visualise the appearance and coverage of biofilm developed on the PWG coupons inserts (Figure 1). The inner surface (insert) of 3 PWG coupons (total area 90 mm^2^) was collected at the end of the experiment (day 28). Coupons inserts were fixed overnight with 5% glutaraldehyde (Glutaraldehyde, 25% aqueous solution, Thermo Fisher United Kingdom) to preserve them until further analysis. Following this, inserts were fixed in 2% aqueous osmium tetroxide for 1 h at room temperature. A series of ethanol dilutions in distilled water were used for dehydrating the inserts in 15-min steps as follows: 75%, 95%, two steps of 100% ethanol, and 100% over anhydrous copper sulphate. The inserts were immersed in a 50/50% (*v*/*v*) solution of absolute ethanol and hexamethyldisilazane for 30 min and then transferred to 100% hexamethyldisilazane for a further 30 min. Samples were mounted onto a pin-stub using a Leit-C sticky tab and Leit-C plast, gold coated using an Edwards S150B sputter coater and examined in using a Tescan Vega3 LMU SEM at The University of Sheffield (UoS).

### Sequencing Analysis of Biofilm Samples

DNA was extracted using a method based on proteinase K digestion followed by a standard phenol/chloroform/isoamyl alcohol extraction (Neufeld et al., 2007). Quantity and purity of the extracted DNA were assessed using NanoDrop ND-1000 spectrophotometer (NanoDrop, Wilmington, DE, United States).

Sequencing of marker genes for bacteria and fungi was performed on biofilm samples (coupons = 30 and sections = 12, total 42 samples) by Illumina MiSeq technology with the paired-end protocol by Mr. DNA Molecular Research Laboratory (^1^ Shallowater, TX, United States) following the manufacturer’s guidelines. For bacterial characterisation, the primers 28F and 519R spanning the V1 to V3 hypervariable regions of the 16S rRNA gene were used. The ITS2 region was used for characterisation of fungal communities. Sequencing data obtained from the 16S rRNA and ITS2 genes (available in^2^, reference: PRJNA656940) was processed by means of Mr. DNA’s analysis pipeline (MR DNA, Shallowater, TX, United States) using the software available in www.mrdnafreesoftware.com. In brief, sequences were depleted of barcodes and primers, then sequences <150 bp, with ambiguous base calls and with homopolymer runs exceeding 6 bp were removed from further analysis. Sequences were denoised and Operational Taxonomic Units (OTUs) generated whilst chimeras were removed. OTUs were defined by clustering at 3% divergence (i.e., 97% similarity cut off). Finally, OTUs were taxonomically classified using BLASTn against a database derived from RDPII^3^ and NCBI^4^.

Alpha-diversity metrics including dominance (uniformity of the community from 0, when all taxa are equally present, to 1, when one taxon dominates the community completely), Shannon Index (diversity, number of different OTUs taking into account their relative abundance) and Chao-1 (richness, number of different OTUs), were calculated at 97% sequence similarity cut off, with the software PAST version 4.0 (Hammer et al., 2001).

Whole metagenomics sequencing was performed using six DNA 28-day old-biofilm samples extracted from pipe sections. Sequencing libraries of the six samples were prepared by Mr. DNA Molecular Research Laboratory using a Kapa HyperPlus Single-Index Adapter Kit with TruSeq adapters (Illumina, San Diego, CA, United States), with sample-specific multiplex adaptor according to the manufacturer’s instructions. The libraries were sequenced using a single lane in a HiSeq2500 System (Illumina). Then, MG-RAST Metagenomics Analysis Server v.4.0^5^ (Keegan et al., 2016) was used to analyse the sequencing reads as described in Douterelo et al. (2018a). Sequence analysis can be found in https://www.mg-rast.org/linkin.cgi?project=mgp93936. In brief, a pre-processing and quality control step was used to removed artificial duplicate replicates (dereplication) according to the method of Gomez-Alvarez et al. (2009) and low quality sequences as described by Cox et al. (2010). To identify genes and their functions, the reads were annotated using the genetic functions using the clusters of orthologous groups of proteins (COGs) database (Jensen et al., 2008). COGs database classifies the sequences in three hierarchy levels (level 1, level 2 and specific functional annotated categories). The higher the level the more specific the annotated functional categories, for example, level 1 has 4 categories (cellular processes, information storage and processing, metabolism, and poorly characterised), but level 2 has 24 categories.

### Statistical Analysis of Microbial Communities

To stablish significant differences in microbial communities (bacteria and fungi, respectively) over time and with phosphate treatment, the relative sequence abundance at 97% sequence similarity cut off of 42 samples was transformed by square root calculations, and Bray–Curtis similarity matrixes were obtained using PAST version 4.0 (Hammer et al., 2001). The results were then visualised with non-metric multi-dimensional scaling (nMDS) diagrams. The analysis of similarity statistics (ANOSIM) was applied to test the significance of the differences between samples using the same Bray–Curtis similarity distance matrixes with the software PAST version 4.0. From the ANOSIM analysis, R statistic values between 0 and 1 indicate the strength of the impact that the factors had on the samples, in this case treatment (control and phosphate treatment) and time (0, 7, 14, 21, 28 days), where 1 indicates high separation of the samples between levels of the factor and 0 indicates no separation (Douterelo et al., 2018b).

To determine if the observed differences in cell counts were significant, pairwise comparisons between samples from different loops and samplings days were made with IBM SPSS 20, using the non-parametric Kruskal-Wallis test and Mann-Whitney *U*-test, respectively. Differences in alpha diversity indices between sampling days, treatments and bacterial and fungi indices were also tested using Mann-Whitney *U*-test.

## Results

### Water Physico-Chemical Analysis

Results from the physico-chemical analysis of the water are shown in Table 1. Most physico-chemical parameters were similar over time among all the samples: water temperature (12.8–14.20°C), pH (4.60–7.00), turbidity (0.13–0.26 NTU) and TOC (1.10–1.67 mg/L). Orthophosphate dosing was effective at keeping stable phosphate levels in the water at 1.05–1.17 mg/L in control condition and 2.4–2.9 mg/L in phosphate treatment over the studied period. Free and total chlorine was slightly higher under phosphate treatment conditions (0.28–0.45 mg/L free Cl_2_ and 0.38–0.64 mg/L total Cl_2_) than in control (0.24–0.41 mg/L free Cl_2_ and 0.35–0.47 mg/L total Cl_2_). Iron and manganese concentrations were under detection range during the experiment.

### Total Cell Counts Obtained by Flow Cytometry

**FIGURE 2:**
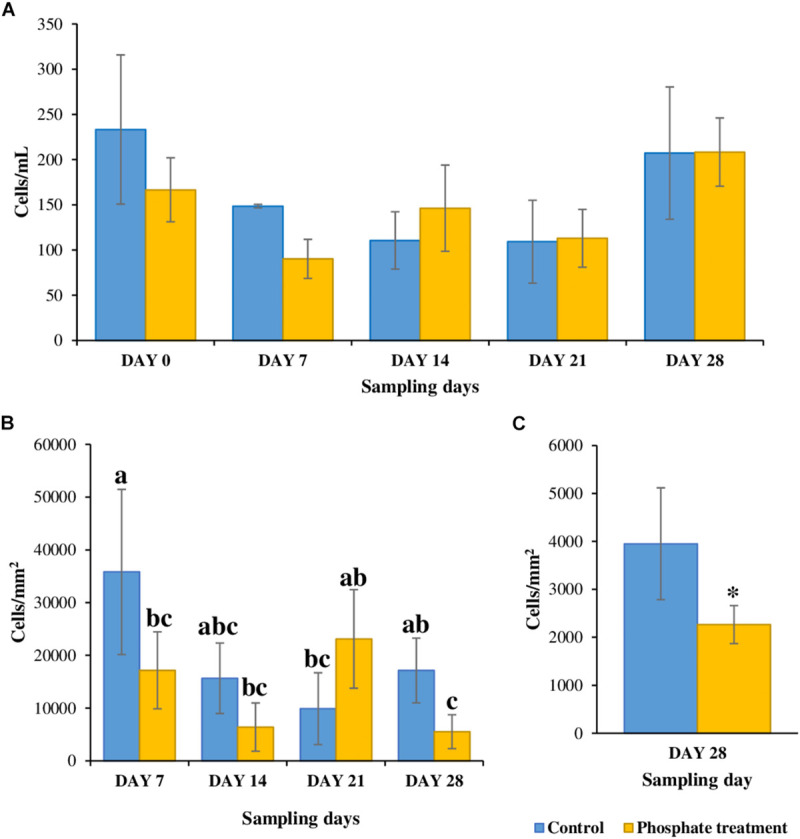
Total cell count at different sampling points. **(A)** Water samples (*n* = 5). **(B)** Biofilm samples from coupon’s inserts (*n* = 3); samples with same letters (a, b or c) represent statistically non-significant samples while samples with different letters are significatively distinct. **(C)** Biofilm samples from sections (*n* = 6), * shows significatively differences.

Results from counting cells in bulk water samples during the experiment (Figure 2A), were highly variable and ranged from 83 to 233 cells/mL of water. No differences were found between control and phosphate-added water samples with reference to the number of cells in all the samples (*p*-value < 0.05).

Regarding biofilms, they tended to present less cells under phosphate-added conditions (5,519–23,117 cells/mm^2^) when compared with control case samples (9,885–35,812 cells/mm^2^), especially in days 7 and 28. Significant differences (*p*-value < 0.05) were found for 28-day-old biofilm samples grown under phosphate treatment when compared with the control, the phosphate-enriched samples had fewer cells in both inserts from PWG coupons and 0.5 m pipe sections (Figures 2B,C) with significant differences (*p*-value < 0.05).

### SEM Analysis of Biofilm Samples

**FIGURE 3:**
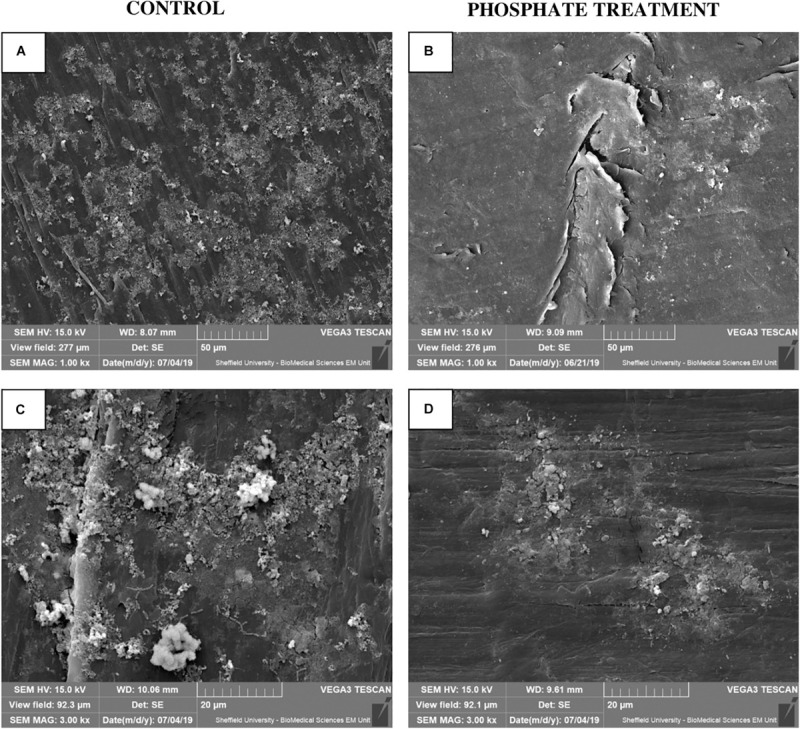
Biofilm micrograph images at 28-day biofilms using SEM (*n* = 3). **(A,C)** show biofilm structure under United Kingdom normal water phosphate concentrations (Control), at 1.00 kx **(A)** and 3.00 kx **(C)** SEM magnification. **(B,D)** show biofilm physical appearance under the effect of 2 mg/l of phosphate (Phosphate treatment) at 1.00 kx **(B)** and 3.00 kx **(D)** SEM magnification.

SEM micrographs (Figure 3) show visual differences in biofilm development on pipe surfaces with regards to phosphate dosing. Biofilm-like structures grown under phosphate control conditions were covering the entire surface of the studied area and were not limited to specific areas (Figures 3A,C). However, under phosphate added conditions, biofilm-like structures tended to accumulate on small/specific areas of the analysed surface, without covering the entire surface of the coupon/insert (Figures 3B,D).

### Analysis of Microbial Communities in Biofilms

#### Bacterial–Fungal Community Structure: Taxonomic Analysis Using Marker Genes

**FIGURE 4:**
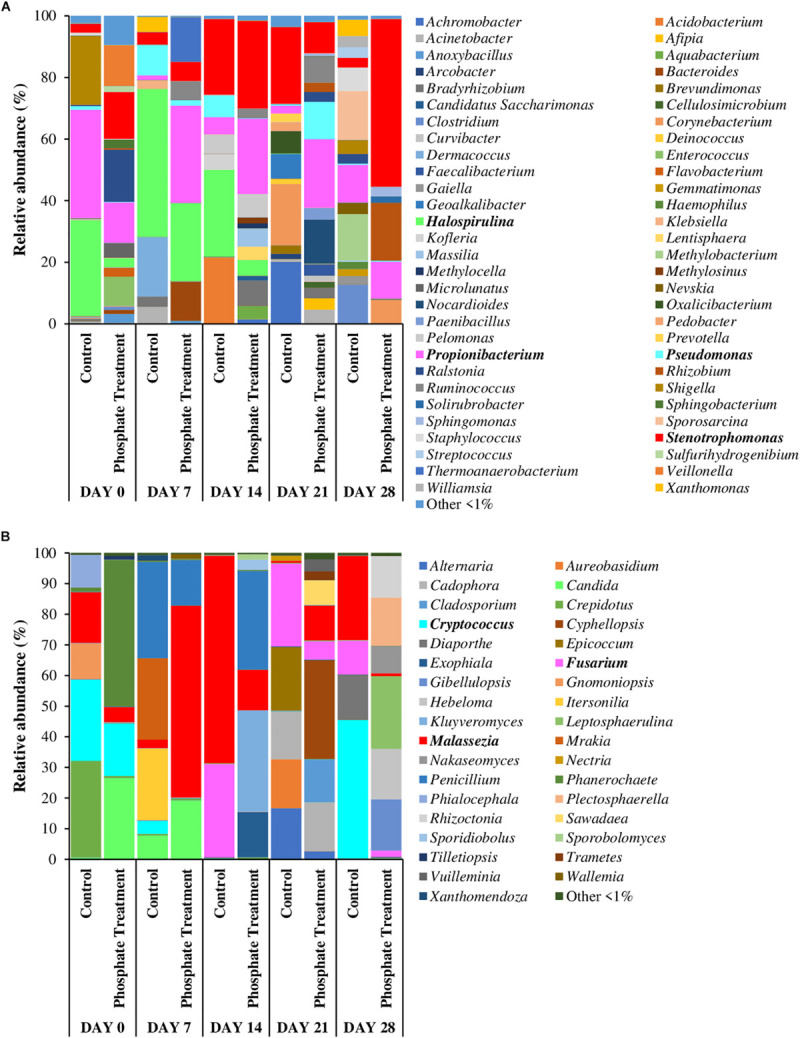
Relative abundance of most abundant bacterial **(A)** and fungal **(B)** genera. Average of relative abundance of the most abundant fungal genera (*n* = 3) in different sampling time (0,7, 14, 21, and 28 days) of different treatments (phosphate treatment and control) belonging to biofilm coupons samples. The most abundant genera are those which have a relative abundance up to 1% in all the samples.

**FIGURE 5:**
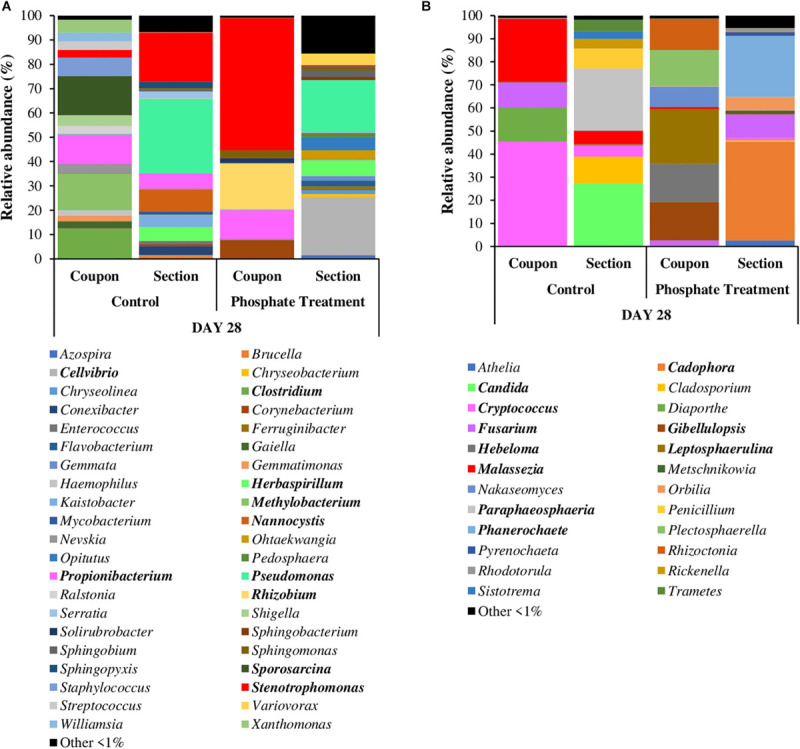
Relative abundance of most abundant bacterial **(A)** and fungal **(B)** genera in coupons and sections. It is shown the average of relative abundance of the most abundant bacterial and fungal genera (*n* = 3) at 28 days of different treatments (phosphate treatment and control) and in coupons and section samples. The most abundant genera are those which have a relative abundance up to 1% in all the samples.

Figures 4, 5 show the relative abundance of bacterial and fungal genera (i.e., 97% sequence similarity cut off) of biofilm developed on PWG coupons and sections. Bacteria belonging to the genera *Stenotrophomonas* (2.8–54.4%) and *Propionibacterium* (1.4–35.1%) were present in all samples, independently of the time and treatment analysed. *Halospirulina* was mainly present in the first 14 days, more abundant in control case (28.2–48.0%) than in phosphate treatment samples (3.1–25.4%). *Pseudomonas* was detected in the biofilm samples during the first 2 weeks of development especially in control samples (1.1–9.9%) and in day 21 of phosphate treatment samples (12.1%). Regarding bacterial communities identified in section and coupons samples in day 28 samples (Figure 5A), it was observed that *Stenotrophomonas* (54.4%), *Rhizobium* (18.8%), *Propionibacterium* (12.0%), and *Corynebacterium* (7.7%) were the genera most represented in the phosphate treatment coupon samples, whilst in section samples, *Cellvibrio* (23.7%), *Pseudomonas* (21.4%), *Herbaspirillum* (6.4%), and *Optitutus* (5.4%) were the predominant genera. Under control conditions, the genera with more representation in coupon samples were *Sporosarcina* (15.9%), *Methylobacterium* (15.0%), *Clostridium* (12.4%), *Propionibacterium* (12.2%), and *Staphylococcus* (7.6%) and in sections *Pseudomonas* (30.7%), *Stenotrophomonas* (20.3%), *Nannocystis* (9.0%), *Propionibacterium* (6.2%), and *Herbaspirillum* (5.7%).

When fungi were analysed, *Malassezia* (0.7–67.7%) showed high relative abundance in all the coupon samples. *Candida* was especially abundant in days 0 and 7 in phosphate treatment samples (7.8–26.5%) and in the control samples obtained on day 7 (19.2%). *Cryptococcus* was highly represented in day 0 samples under both treatment cases (17.2% under phosphate treatment and 26.5% in control) and then again at 28 days in control samples (45.2%). *Fusarium* was present in the samples from the last 2 weeks of the experiment (i.e., days 14 to 28), particularly in control case samples (11.0–30.4%). Some of the most abundant fungal genera in 28-day-old coupon samples under phosphate treatment were *Leptosphaerulina* (23.8%), *Gibellulopsis* (16.7%), *Hebeloma* (16.5%), *Plectosphaerella* (15.8%), and *Rhizoctonia* (13.6%), whilst in control samples *Cryptococcus* (45.2%), *Malassezia* (27.5%), *Diaporthe* (14.6%), and *Fusarium* (11.0%) were highly represented. In section samples (Figure 5B), *Cadophora* (42.9%), *Phanerochaete* (26.6%), and *Fusarium* (10.1%) were the most abundant genera under phosphate treatment, and *Candida* (27.1%), *Paraphaeosphaeria* (26.9%), *Cladosporium* (11.6%), and *Penicillium* (8.7%) in control case samples.

#### nMDS Analysis of Microbial Communities (Fungi vs. Bacteria)

**FIGURE 6:**
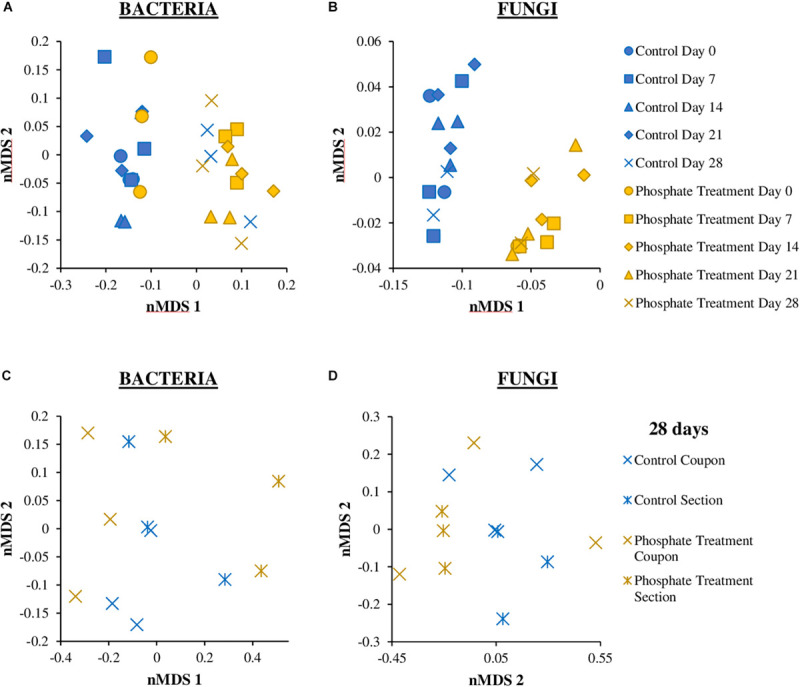
Non-metric multidimensional scaling (nMDS) plot based on the bacterial **(A,C)** and fungal **(B,D)** relative abundance of biofilm coupons **(A,B)** and section **(C,D)** samples. The analysis was based on Bray–Curtis similarity matrix calculated from the relative abundance of bacteria and fungi OTUs at 97% cut off. The different groups are clustering according to the treatment used (Phosphate treatment and Control) and the time sampling (0, 7, 14, 21, 28 days).

The distribution of microbial communities in nMDS graphs (Figure 6), showed that the bacterial community structure were similar between the control and the phosphate treated samples (Figures 6A,C), particularly in samples from day 0 and day 28. The ANOSIM analysis did not indicate any significant differences in the distribution of the communities between the two experimental conditions (control and phosphate treatment) (ANOSIM: Global *R* = 0.0188 *p*-value = 0.327) and between the different support materials (coupons and sections) (Global *R* = 0.1821 *p*-value: 0.017). With respect to fungal communities (Figures 6B,D), significant differences were found in the distribution of the communities between the two conditions studied (ANOSIM: Global *R* = 0.2913 *p*-value = 0.004). However, no significant differences were observed between samples collected at different support materials for both treatments (ANOSIM: Global *R* = 0.1235 *p*-value = 0.157).

#### Alpha Diversity Analysis of Biofilm Microbial Communities

Results from alpha diversity analysis of biofilm communities were detailed in Supplementary Figure 1. Significant differences between control and phosphate treatment samples were found for bacterial dominance between 0 and 28- day-old samples and for richness index values between 7- and 14-day-old samples (*p* value < 0.05). The richness index values were slightly higher for control samples when compared with phosphate treated samples over the whole experimental period, and dominance decreased considerably in 28-day old control samples. When temporal variation was analysed in both conditions; bacterial diversity and richness tended to increase in biofilms over time, whilst bacterial dominance decreased.

When fungal alpha diversity was analysed, no significant differences were detected for any sample (*p*-value > 0.05). The indices did not show significant variations between both conditions (control and phosphate treatment), and they tended to be stable during the duration of the experiment. The richness index increased lightly over time for both experimental conditions.

When bacterial and fungal alpha diversity was compared, only significant differences were found in richness at day 7 and day 21 in control samples, and in day 7 in phosphate treatment samples (*p*-value < 0.05). In summary, fungal communities were more dominant and bacterial communities were more diverse and richer over time.

### Whole Metagenomics Analysis: Functional Genetic Analysis

**FIGURE 7:**
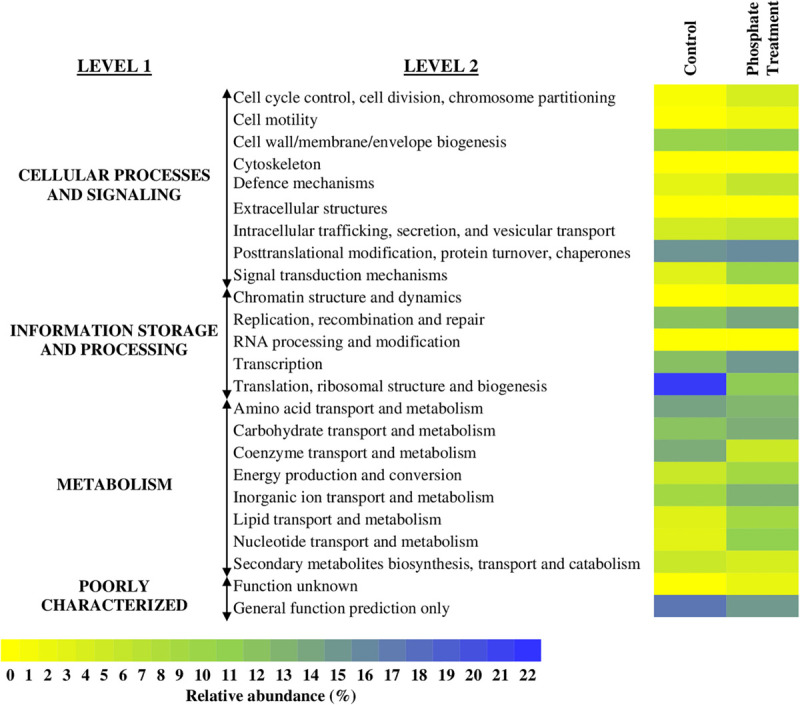
Heatmap with the relative abundance of annotated genes in the whole metagenome of each of the sequenced samples. It is shown MG-RAST analyses of biofilm samples based in COGs database. The average of relative abundance (*n* = 3) of the level 1 and 2 functional categories is expressed in a rage of colour from yellow (0%) to blue (22%).

The relative abundances of annotated functional genes are detailed in Figure 7 and Supplementary Figure 2. The functional categories that showed higher percentages of relative abundance at functional level 2 were: translational ribosomal structure and biogenesis (control: 21.2%, phosphate treatment: 4.7%), post-translational modification, protein turnover and chaperones (control: 11.05%, phosphate treatment: 11.9%), transcription (control: 6.1%, phosphate treatment: 10.7%), replication, recombination and repair (control: 5.8%, phosphate treatment: 9.0%), and transport and metabolism of amino acids (control: 9.3%, phosphate treatment: 7.2%).

Further information on specific functional annotated categories in the samples is detailed in Supplementary Figure 2. In the control case samples, most of the sequences annotated were related to the metabolism of nitrogen (mainly synthesis of amino acids), ribosomal proteins, and several transmembrane proteins. For phosphate treatment samples, most of the sequences were related to ATPases, ion transporters, and DNA-interact proteins.

Despite that the effect of phosphate on biofilm communities was the main aim of this study, it must be noted that the sequences related to phosphate transport and metabolism were not highly represented in the functional analysis. Functional categories associated with phosphate did not change with the addition of this chemical, only a predicted ATPase related to phosphate starvation-inducible protein PhoH was considerable represented in the analysis of phosphate treatment samples (1.14%).

## Discussion

### Effect of Phosphate on Water Quality Characteristics

No significant changes were found in water physico-chemical characteristics over time and between treatments (Table 1), given that the same water source and pipe material were used for the experiments. Chlorine concentration increased slightly under phosphate dosing conditions, nevertheless, we consider that these differences are not enough to cause a significant change in the microbial communities of the biofilm. This statement is done basing on a similar research performed in the same facility (Fish and Boxall, 2018) which studied the impact of free chlorine concentration on the DWDS biofilm microbiome and it was necessary using extremely different chlorine concentrations (0.03 ± 0.05, 0.45 ± 0.05, and 0.80 ± 0.16 mg/L) to observed significant differences in microbial biofilm communities at 28-days.

### Effect of Phosphate Treatment on Biofilm Structure

In this study, biofilms obtained from phosphate treatment samples showed less total cells (Figure 2) and restricted/limited development on coupon surfaces when they were observed by SEM (Figure 3). Contrary to these results, other studies in drinking water related systems, reported an increase of cells in biofilms developed under different phosphate treatments (Fang et al., 2009; Noh et al., 2019). An explanation for these contradictory results might be that these studies were performed using annular bioreactors (Fang et al., 2009) and biological activated carbon columns (Noh et al., 2019) where biofilms were less exposed to hydraulic forces than under representative hydraulic conditions in DWDS. Besides, previous studies have shown that the addition of phosphate in drinking water can reduce the synthesis of EPS by biofilms (Fang et al., 2009; Kirisits et al., 2013; Liu et al., 2016; Noh et al., 2019). The EPS matrix, among other functions, contribute to cell adhesion and anchoring, stability of aggregates and films, as well as physical and chemical protection (Neu and Lawrence, 2010) against antibiotics (Mah and O’Toole, 2001), and chlorine (Chen and Stewart, 1996). Therefore, microorganisms grown under higher phosphate conditions might produce less EPS, thus favouring a weaker biofilm structure, with less capacity of adhesion and more sensitive to external forces and chemical agents. This could explain the results from this study, where a lower number of cells was observed in biofilms exposed to phosphate treatment. Considering this results, phosphate can promote structurally weaker biofilms that can be easily removable and mobilised into the bulk water, but increasing the risk of discolouration and the presence of opportunistic pathogens at tap level (Douterelo et al., 2013).

### Effect of Phosphate on the Biofilm Taxonomic Analysis

According to the results found in this experiment, there were not significant differences between the bacterial communities in the two experimental conditions (Figures 6A,C). However, the increase in phosphate tended to reduce bacterial richness, whilst promoted bacterial dominance, thus fewer OTUs were predominant in the community. This finding contradicts those of Payne et al. (2016), who observed an increase in size and diversity of biofilm communities as a result of phosphate treatment in galvanic macrocells with lead and copper components fed with drinking water. Similarly, Jang et al. (2012), reported an increase of species diversity in biofilm when phosphate was added under low residual chlorine conditions in an annular reactor also fed with drinking water. Despite phosphate has been considered one of the main factors that can affect bacterial distribution in DWDS (Douterelo et al., 2016a), it is possible that the doses used in this study were not high enough to trigger a significant change in the communities or that phosphate is not a limiting factor for bacterial growth.

Nevertheless, phosphate did affect the structure of fungal communities (Figure 6B), suggesting that this chemical can be a limited nutrient for these organisms in DWDS. Little is known about phosphate effect on fungal populations in DWDS, but fungi are known to form symbiotic associations with most plants and release mineral nutrients, specially phosphate (Smith and Read, 2008). In this research, under both experimental conditions, we found several bacterial and fungal genera with members able to solubilize and accumulate phosphate. For example, bacteria like *Acinetobacter*, *Herbaspirillum*, *Pseudomonas*, *Rhizobium*, *Klebsiella* or *Xanthomonas*, and some fungi including *Alternaria*, *Cladosporium*, *Penicillium* or *Fusarium* have been identified as phosphate solubilizers (Sidat et al., 1999; Estrada et al., 2013; Sharma et al., 2013; Alori et al., 2017; Saritha and Prasad Tollamadugu, 2019). However, the addition of phosphate did not change significatively the relative abundance of these type of microorganisms in biofilm samples, reinforcing the idea that phosphate in this study was not a limiting factor for microbial growth and/or that the experimental dosing was not high enough to promote any important changes.

### Effect of Phosphate in Genetic Functional Traits

In this study, the whole metagenomics data showed differences in the most represented functional traits between the two phosphate conditions tested (Supplementary Figure 2). In control samples, sequencing reads associated to nitrogen metabolism were predominant, whilst sequences related to ATPases, ion transporters, and DNA-interact proteins were highly abundant under phosphate treatment. This suggests that the addition of phosphate might change the genetic composition of the biofilm communities, thus affecting functional traits. The high relative abundance of sequences related to nitrogen metabolism within biofilms in this study was not expected, since autotrophic nitrifying organisms (bacteria and archaea) are highly found within chloraminated DWDS (Wilczak et al., 1996), where their growth is promoted by ammonia availability (Potgieter et al., 2020).

In relation to phosphorus metabolism, sequences related with “luxury uptake of phosphorus/phosphate,” like polyphosphate kinase, were found in samples from phosphate treatment. Luxury uptake is a phenomenon that enables bacteria to accumulate phosphate as PolyP granules when phosphate is in excess and/or under amino acid starvation conditions, even though it might not be favourable for their growth (Harold, 1966; Kato et al., 1993; Kuroda et al., 1997; Khoshmanesh et al., 2002; Hirota et al., 2010). It must be noted that in this study both the relative abundance of phosphate metabolism related sequences and the presence of microorganisms able to accumulate phosphate was low. Despite that the phosphate concentrations used in this study did not trigger important functional changes, longer dosing periods (i.e., >28 days) may favour the presence of microorganisms with high affinity to phosphate, as some authors have previously suggested (Appenzeller et al., 2001). Similarly, research based on gene expression using RNA sequencing instead of DNA would help future studies to identify specific active functional traits involved in phosphate metabolism.

## Conclusion

This study has yielded new information regarding the impact of phosphate dosing to control plumbosolvency on biofilms within DWDS. The following conclusions can be drawn as a result of the research:

1. Increasing the phosphate dose triggered a reduction in biofilm cell numbers and promoted a less consolidated and poorly distributed biofilm structure.
2. Phosphate enrichment in drinking water did not significantly affected bacterial community structure, while influenced the composition of fungal communities.
3. Bacterial communities were more diverse and richer over time when compared with fungal communities.
4. Sequences related to nitrogen metabolism (mainly in amino acids synthesis) and ribosomal proteins were predominant in control samples, whilst phosphate enrichment promoted the presence of sequences related to ATPases, ion transporters and DNA-interacting proteins.

## Data Availability Statement

The datasets presented in this study can be found in online repositories. The names of the repository/repositories and accession number(s) can be found below: https://www.ncbi.nlm.nih.gov/, PRJNA656940.

## Author Contributions

ER, GD, and ID were involved in the design of the experiment. ER, GD, and CC participated in the execution of the experiments. ER extracted the DNA from samples and did the SEM micrographs. ER and GD performed the flow cytometry analysis and analysed the results. ER, GD, and ID elaborated the manuscript. CC participated in the corrections of the manuscript. All authors contributed to the article and approved the submitted version.

## Conflict of Interest

The authors declare that the research was conducted in the absence of any commercial or financial relationships that could be construed as a potential conflict of interest.

## Footnotes

1 www.mrdnalab.com

2 https://www.ncbi.nlm.nih.gov/sra/PRJNA656940

3 http://rdp.cme.msu.edu

4 www.ncbi.nlm.nih.gov

5 http://metagenomics.anl.gov
