# Supplementary material for: Phosphate Dosing in Drinking Water Distribution Systems Promotes Changes in Biofilm Structure and Functional Genetic Diversity
Source: Front Microbiol. 2020 Dec 17;11:599091. doi: 10.3389/fmicb.2020.599091 (PMC7773730; doi:10.3389/fmicb.2020.599091)
Supplement: Supplementary file 1 [file Image_1.pdf]

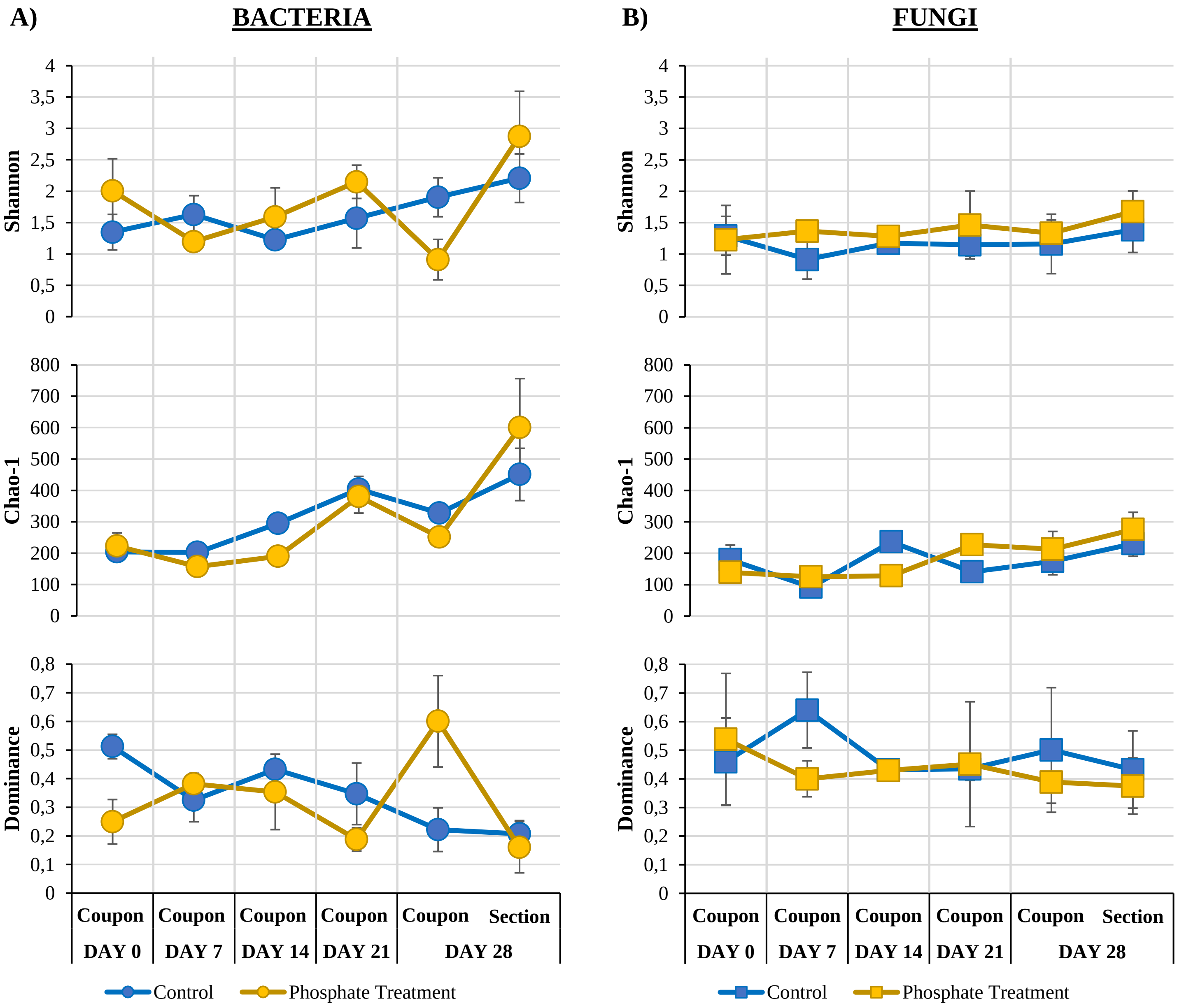

Supplementary Figure 1: Dominance, Shannon (Diversity index) and Chao I (richness index) for biofilm bacterial (A) and fungal (B) OTUs at 97 % cut off. It is shown diversity indices average with standard errors (n=3) for bacteria and fungus under different treatments (Phosphate treatment and Control) and different support materials (coupons and sections).
