# Supplementary material for: Phosphate Dosing in Drinking Water Distribution Systems Promotes Changes in Biofilm Structure and Functional Genetic Diversity
Source: Front Microbiol. 2020 Dec 17;11:599091. doi: 10.3389/fmicb.2020.599091 (PMC7773730; doi:10.3389/fmicb.2020.599091)
Supplement: Supplementary file 2 [file Image_2.pdf]

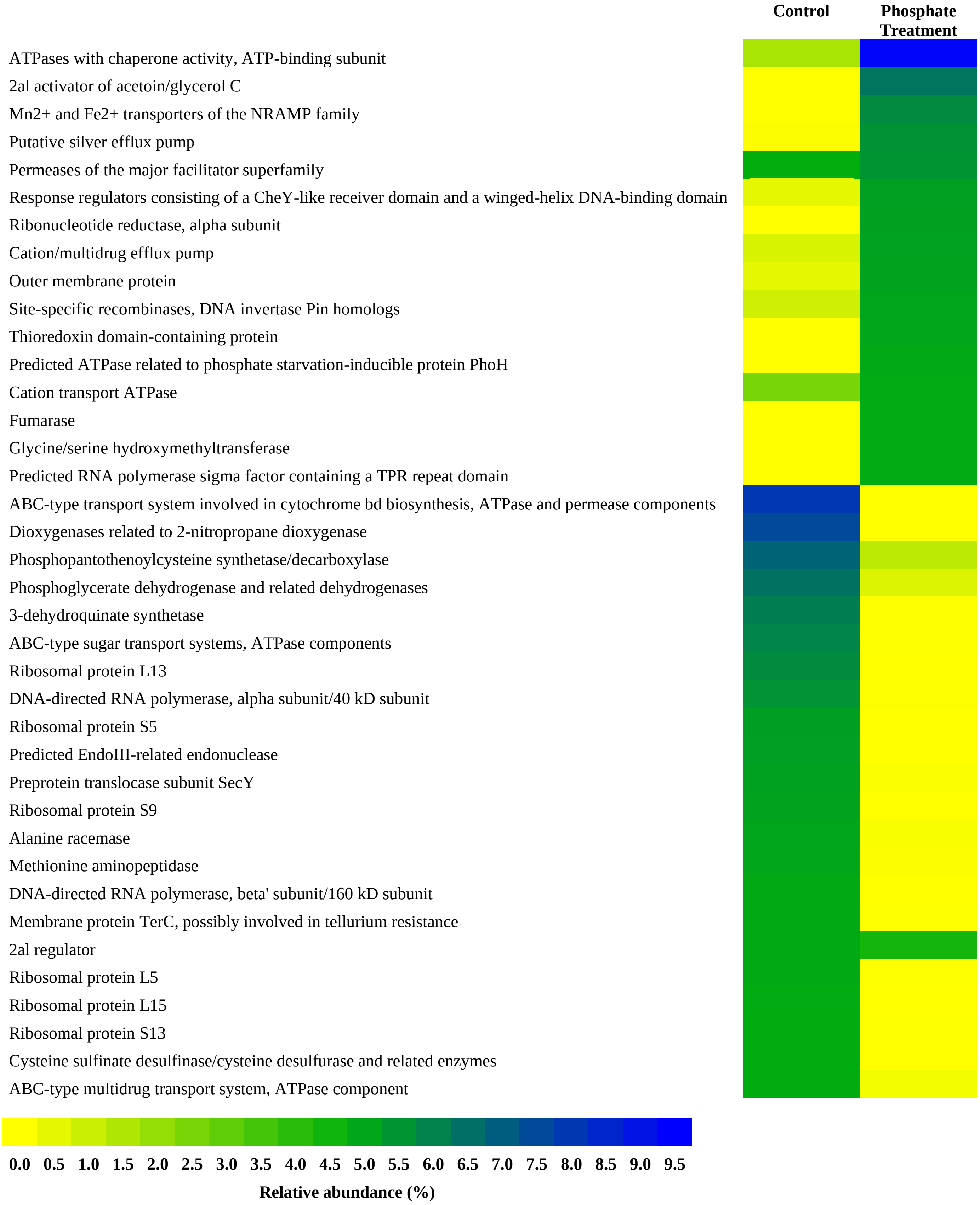

Supplementary Figure 2: Relative abundance of specific functional annotated categories in samples from control and phosphate treatment cases with a high proportion. *It is shown the average of n = 3 in each studied case.*
